# Dynamic Metabolic Parameters and Deauville Score on 18F-FDG PET/CT for Prognostic Assessment in Pediatric Burkitt Lymphoma

**DOI:** 10.3390/children13091162

**Published:** 2026-08-28

**Authors:** Emine Goknur Isik, Sifa Sahin, Duygu Has Simsek, Ebru Yilmaz, Sema Buyukkapu Bay, Rejin Kebudi, Seher Nilgun Unal, Hikmet Gulsah Tanyildiz

**Affiliations:** 1 Department of Nuclear Medicine, Istanbul Faculty of Medicine, Istanbul University, 34093 Istanbul, Türkiye; d.hassimsek@istanbul.edu.tr (D.H.S.);; 2 Department of Pediatric Hematology and Oncology, Faculty of Medicine, Afyonkarahisar Health Sciences University, 03200 Afyonkarahisar, Türkiye; 3 Department of Nuclear Medicine, Istanbul Florence Nightingale Hospital, 34381 Istanbul, Türkiye; 4 Department of Pediatric Hematology and Oncology, Oncology Institute, Istanbul University, 34093 Istanbul, Türkiyerejin.kebudi@istanbul.edu.tr (R.K.); gulsahtanyildiz@istanbul.edu.tr (H.G.T.)

**Keywords:** burkitt lymphoma, pediatric oncology, 18F-FDG PET/CT, deauville score, metabolic tumor volume, total lesion glycolysis

## Abstract

**Highlights:**

**What are the main findings?**

- The %ΔSUVmax and the end-of-treatment Deauville score predicted survival in pediatric Burkitt lymphoma.
- Baseline SUVmax, MTV, and TLG showed only modest, estimated prognostic value in this cohort.

**What are the implications of the main findings?**

- Dynamic metabolic response may be a more reliable early prognostic marker than baseline tumor burden in pediatric BL.
- A %ΔSUVmax reduction of ≥73.6% may help identify patients at lower risk of relapse or death after treatment.

**Abstract:**

**Background/Objectives:** 18F-FDG PET/CT is increasingly used in pediatric Burkitt lymphoma (BL) for staging and treatment response assessment, but data on quantitative prognostic markers remain limited. **Methods:** We retrospectively evaluated 33 children with pathologically confirmed BL who underwent baseline and end-of-treatment (EOT) 18F-FDG PET/CT between 2009 and 2024. SUVmax, metabolic tumor volume (MTV), and total lesion glycolysis (TLG) were measured at baseline and EOT, and the Deauville score (DS) was assigned visually. Progression-free survival (PFS) and overall survival (OS) were estimated with the Kaplan–Meier method; receiver operating characteristic (ROC) analysis identified predictive thresholds. **Results:** Three-year and 5-year OS was 89.6%, and PFS was 90.1%. The percentage reduction in SUVmax from baseline to EOT (%ΔSUVmax) was the strongest predictor of survival (AUC = 0.94; optimal cutoff ≥ 73.6% reduction; sensitivity 100%, specificity 93%). Patients with an unfavorable EOT DS (4–5) had significantly lower OS than those with a favorable DS (1–3) (60.0% vs. 96.4%; log-rank *p* = 0.009). Baseline SUVmax, MTV, and TLG did not differ significantly by stage or splenic involvement. Given the small number of survival events (*n* = 3), the corresponding hazard ratios and confidence intervals should be regarded as exploratory and hypothesis-generating rather than definitive effect sizes. **Conclusions:** Dynamic metabolic response such as %ΔSUVmax and the EOT Deauville score are promising prognostic markers in pediatric BL. Given the small number of events, these findings warrant validation in larger, prospective cohorts.

## 1. Introduction

Lymphoma is the third most common cancer in children, and non-Hodgkin lymphoma (NHL) accounts for approximately half of pediatric lymphomas [1]. Burkitt lymphoma (BL), a high-grade subtype of NHL, typically affects children and young adults; sporadic BL is the most common variant in the Western world and is associated with Epstein–Barr virus infection in 25–40% of cases. The abdomen is the most common presentation site. BL is highly chemo-sensitive, with survival rates ranging from 50% to over 80% depending on stage and age at diagnosis [1,2,3,4,5]. Despite its aggressive biology and high 18F-fluorodeoxyglucose (18F-FDG) avidity, data on predictive prognostic markers in pediatric BL remain limited.

Early and accurate staging is essential for guiding the intensive chemotherapy regimens used in BL. 18F-FDG PET/CT has become increasingly incorporated into pediatric lymphoma imaging protocols, improving diagnostic accuracy relative to conventional imaging and contributing to staging, treatment response monitoring, and detection of residual or relapsed disease; pediatric-specific series have also described occasional pitfalls, including false-positive post-chemotherapy uptake related to inflammatory or reactive changes [6,7]. For response assessment, the five-point Deauville score (DS), which compares lesion uptake with mediastinal and hepatic blood-pool activity, was proposed at the International Workshops on interim PET in lymphoma [8] and has been incorporated into the Lugano response criteria for Hodgkin lymphoma and high-grade NHL such as diffuse large B-cell lymphoma (DLBCL) and follicular lymphoma [9,10]. Semiquantitative and quantitative PET parameters such as maximum standardized uptake value (SUVmax), metabolic tumor volume (MTV), and total lesion glycolysis (TLG) have also been studied in staging and response assessment in both pediatric and adult BL [11,12,13,14,15].

A negative EOT scan by DS has been linked to more favorable outcomes in both adult and pediatric BL: in a French cohort of 19 children treated per the LMB2001 protocol, a negative EOT DS carried a negative predictive value of 93% and was associated with significantly higher 5-year PFS, and comparable associations have been reported in adult series [13,14]. Because SUVmax can be influenced by several technical and biological variables [16], MTV and TLG, which capture both the extent and metabolic intensity of disease, may provide complementary prognostic information. These volumetric parameters have been studied in other FDG-avid lymphomas, including Hodgkin lymphoma and DLBCL [12,17,18,19], and a recent 68-patient pediatric BL cohort found baseline tMTV and TLG, but not SUVmax, to be independent predictors of PFS and OS on multivariate Cox regression [15]. However, to our knowledge no prior study has evaluated the percentage change in MTV and TLG (%ΔMTV, %ΔTLG) between baseline and EOT imaging specifically in pediatric BL, nor combined this with %ΔSUVmax and the EOT Deauville score in the same cohort; a comparable dynamic MTV/TLG analysis has previously been reported in an adult BL cohort [12].

In this study, we aimed to evaluate the contribution of visual (Deauville score), semiquantitative (SUVmax), and volumetric (MTV, TLG) 18F-FDG PET/CT parameters, including their percentage change from baseline to EOT, in predicting treatment response and survival in pediatric BL.

## 2. Materials and Methods

### 2.1. Patients

This single-center retrospective cohort study was approved by the institutional ethics committee (approval date: 5 April 2024; no. 2024/719), and written informed consent was obtained from all patients or their legal guardians. We reviewed the records of patients under 18 years of age with a pathologically confirmed diagnosis of BL who underwent 18F-FDG PET/CT at our institution between April 2009 and 2024. Thirty-three patients had baseline staging and EOT 18F-FDG PET/CT available and were included in the final analysis; 19 of these patients additionally underwent an interim PET/CT during treatment. In total, 33 patients and 85 18F-FDG PET/CT scans were analyzed.

Clinical staging followed the St. Jude NHL staging classification, and patients were grouped into early (stage I–II) and advanced (stage III–IV) disease. Medical records and pathology reports were reviewed for epidemiological features (age at diagnosis, sex), morphological features (nodal versus extranodal involvement, splenic involvement), and metabolic parameters (SUVmax, MTV, TLG, and their absolute and percentage change from baseline to EOT). Treatment and follow-up data, from baseline imaging to last visit or death, were collected for all patients; all patients were treated by the Department of Pediatric Hematology and Oncology at our institution according to the NHL-BFM protocol.

This article is a revised and expanded version of a paper entitled “Evaluation of Quantitative Parameters in F-18-FDG PET-CT for Treatment Response in Pediatric Burkitt Lymphoma” which was presented at the 32nd National Nuclear Medicine Congress, (Belek-Antalya/November 2020), Turkiye [20].

### 2.2. 18F-FDG PET/CT Acquisition and Image Interpretation

Patients fasted for a minimum of 6 h before tracer injection, with a blood glucose threshold of 200 mg/dL. PET/CT examinations were acquired using one of two dedicated scanners available at our institution during the study period: a Siemens Biograph TruePoint HD LSO (Siemens Healthineers, Erlangen, Germany) during the earlier period and a GE Discovery IQ (GE Healthcare, Milwaukee, WI, USA) following the institutional scanner transition in 2018, with Q.Clear reconstruction (β = 300) used for the latter. Imaging was performed in accordance with the pediatric imaging guidelines of the European Association of Nuclear Medicine (EANM) [21]; no oral or intravenous contrast agents or bowel preparation were used.

Images were reviewed on a dedicated workstation (Advantage Workstation 4.7, GE Healthcare) by two board-certified nuclear medicine physicians, who evaluated all pathological lymph nodes and extranodal sites in consensus. The lesion with the highest 18F-FDG uptake at baseline was identified as the reference lesion for SUVmax; this same anatomical location was then re-measured on interim and EOT PET/CT to derive the paired SUVmax values used to calculate %ΔSUVmax, with background SUVmax recorded at that location when no residual FDG-avid lesion was present. SUVmax, MTV, and TLG were measured at baseline, interim (when available), and EOT PET/CT using a semi-automated threshold of 41% of SUVmax, per EANM procedure guidelines [22]. Total MTV summed the volumes of all nodal and extranodal lesions, and each lesion’s MTV was multiplied by its mean SUV and summed across lesions to obtain TLG. The percentage change in each parameter from baseline to EOT (%ΔSUVmax, %ΔMTV, %ΔTLG) was calculated as [(baseline − EOT)/baseline] × 100.

Visual response was assessed using the 5-point DS: 1, no uptake above background; 2, uptake ≤ mediastinum; 3, uptake between mediastinum and liver; 4, uptake moderately increased above liver; 5, markedly increased uptake above liver and/or new lesions. DS 1–3 was considered a negative (favorable) scan and DS 4–5 a positive (unfavorable) scan, consistent with the Lugano classification [9,10,23].

### 2.3. Statistical Analysis

Continuous variables are presented as mean ± SD and range; categorical variables as counts and percentages. Group comparisons of baseline PET parameters by stage and splenic involvement used the Mann–Whitney U test. PFS was defined as time from baseline PET/CT to relapse, progression, death, or last follow-up; OS as time to death from any cause or last follow-up. Both were estimated using the Kaplan–Meier method, with subgroups (favorable vs. unfavorable EOT DS) compared by the log-rank test. ROC analysis assessed the discriminative performance of baseline and %Δ metabolic parameters for PFS/OS events, with cutoffs selected via the Youden index. Univariate Cox regression estimated hazard ratios (HRs) with 95% CI for the strongest candidate predictors; a multivariate model was not performed given only three events. A sensitivity analysis excluding two patients with atypical EOT metabolic burden assessed the robustness of the survival estimates. Two-sided *p* < 0.05 was considered statistically significant. Analyses were performed using Python (version 3.12.3; Python Software Foundation, Wilmington, DE, USA).

## 3. Results

### 3.1. Patient Characterization

Of the 33 patients, 24 (72.7%) were male and nine (27.3%) were female, with a mean age at diagnosis of 10.4 ± 4.7 years (range, 3–18). Median follow-up was 44.0 months (mean 61.9 ± 58.1; range, 4–200). According to the St. Jude classification, three patients (9.1%) had stage II, 10 (30.3%) had stage III, and 20 (60.6%) had stage IV disease; no patient had stage I disease, and 30 (90.9%) presented with advanced-stage (III–IV) disease. Disease distribution was mixed nodal/extranodal in 21 patients (63.6%), purely nodal in nine (27.3%), and purely extranodal in three (9.1%). Splenic involvement was present in three patients (9.1%), two with stage III and one with stage IV disease. Bone marrow involvement was documented in 10 patients (30.3%); all patients with bone marrow involvement had stage IV disease. Patient characteristics are summarized in Table 1.

### 3.2. Baseline PET/CT Findings

Baseline PET/CT was positive in all 33 patients. Mean baseline SUVmax, MTV, and TLG were 17.9 ± 7.5 (range, 4.5–37.4), 691.0 ± 820.4 cm^3^ (range, 17.0–3309.7), and 4600.0 ± 4196.5 g (range, 40.5–17,370.0), respectively. None of the baseline parameters differed significantly between early- and advanced-stage disease (SUVmax, *p* = 0.977; MTV, *p* = 0.382; TLG, *p* = 0.348) or by splenic involvement (SUVmax, *p* = 0.745; MTV, *p* = 0.491; TLG, *p* = 0.382) (Table 2). In contrast, baseline MTV and TLG were significantly higher in the 10 patients with bone marrow involvement than in those without (MTVb: median 923.5 vs. 188.6 cm^3^, *p* = 0.009; TLGb: median 6325.2 vs. 2360.2 g, *p* = 0.020), while baseline SUVmax did not differ by bone marrow status (*p* = 0.769). Three-year OS and PFS were lower in patients with bone marrow involvement (71.4% and 75.0%, respectively) than in those without (95.2% for both), though these differences did not reach statistical significance (log-rank *p* = 0.122 for OS, *p* = 0.132 for PFS); on univariate Cox regression, bone marrow involvement was associated with a numerically increased hazard for a PFS event that did not reach significance (HR 5.26, 95% CI 0.47–58.4; *p* = 0.177).

### 3.3. End-of-Treatment PET/CT Findings

**Figure 1:**
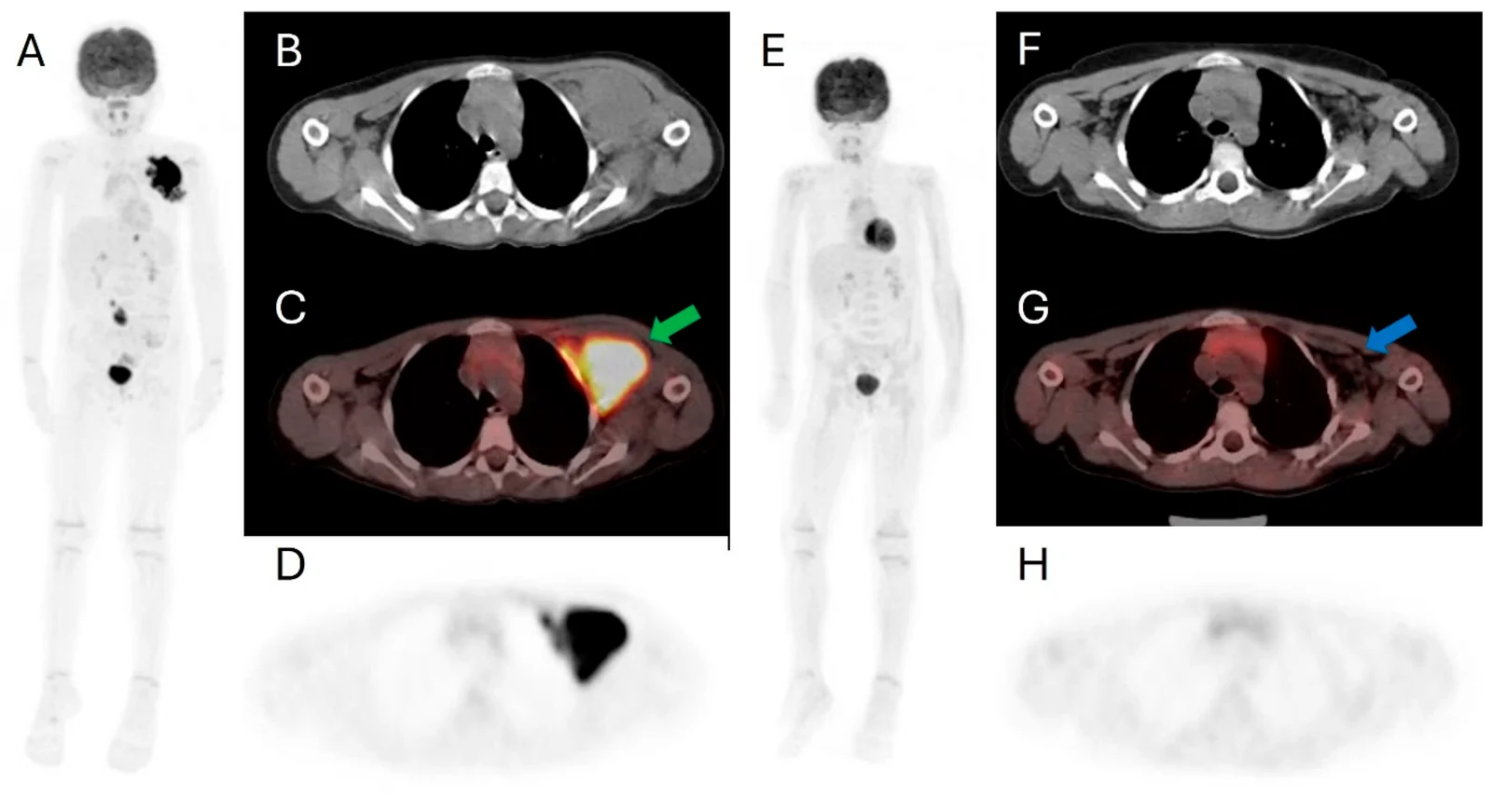
A 6-year-old boy with stage III pediatric Burkitt lymphoma involving the left axilla. (**A**) Baseline whole-body MIP showing an intensely hypermetabolic left axillary mass, with additional foci below the diaphragm (abdominal/pelvic region) consistent with subdiaphragmatic nodal involvement and the patient’s stage III classification. (**B**) Baseline axial CT showing the corresponding left axillary soft-tissue mass. (**C**) Baseline axial fused PET/CT confirming intense FDG uptake within the mass (SUVmax 13.8; green arrow). (**D**) Baseline axial PET image showing the corresponding hypermetabolic focus. (**E**) End-of-treatment whole-body MIP showing resolution of the previously FDG-avid mass. (**F**) End-of-treatment axial CT showing near-complete resolution of the soft-tissue mass. (**G**) End-of-treatment axial fused PET/CT showing only background-level activity at the site of the prior lesion (blue arrow), corresponding to a Deauville score of 2. (**H**) End-of-treatment axial PET image showing no residual abnormal uptake. Baseline MTV, TLG, and SUVmax were 82.2 cm^3^, 723.3 g, and 13.8, respectively; corresponding end-of-treatment values were 15.9 cm^3^, 10.4 g, and 1.1, representing a 92.0% reduction in SUVmax, consistent with the study cutoff (≥73.6%) for favorable metabolic response.

At EOT, mean SUVmax, MTV, and TLG decreased to 2.5 ± 0.9 (range, 0.5–5.0), 87.0 ± 163.7 cm^3^ (range, 1.3–735.8), and 161.8 ± 351.0 g (range, 1.9–1602.6), respectively. The mean percentage reduction from baseline to EOT was 83.3 ± 10.7% for SUVmax, 82.9 ± 15.2% for MTV, and 95.5 ± 9.4% for TLG (Table 3). Interim PET/CT (available in 19 patients) showed mean SUVmax, MTV, and TLG of 3.2 ± 1.6, 82.2 ± 178.8 cm^3^, and 144.7 ± 281.5 g, respectively (Figure 1).

### 3.4. Treatment Response Evaluation

At EOT, the DS distribution was DS1 in eight patients (24.2%), DS2 in 12 (36.4%), DS3 in eight (24.2%), DS4 in three (9.1%), and DS5 in two (6.1%); 28 patients (84.8%) had a favorable (DS1–3) and five (15.2%) an unfavorable (DS4–5) EOT scan. Nineteen of the 33 patients underwent an additional interim PET/CT during treatment. Three patients (9.1%) died during follow-up: two due to treatment-related complications during chemotherapy, and one due to disease progression following relapse.

### 3.5. Survival Analysis

**Figure 2:**
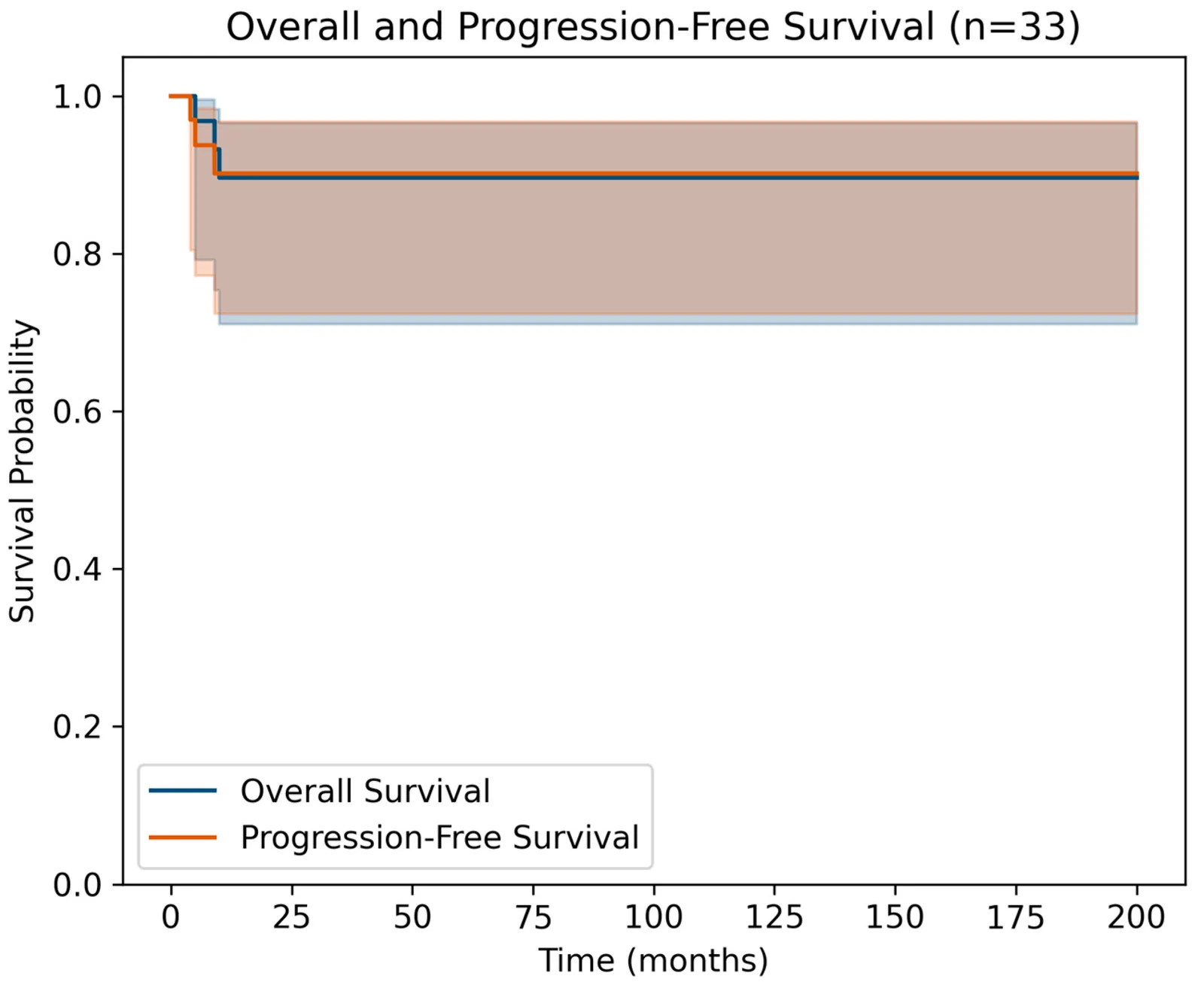
Kaplan-Meier estimates of overall survival (OS) and progression-free survival (PFS) for the full cohort (*n* = 33). Shaded bands represent 95% confidence intervals for each curve.

**Figure 3:**
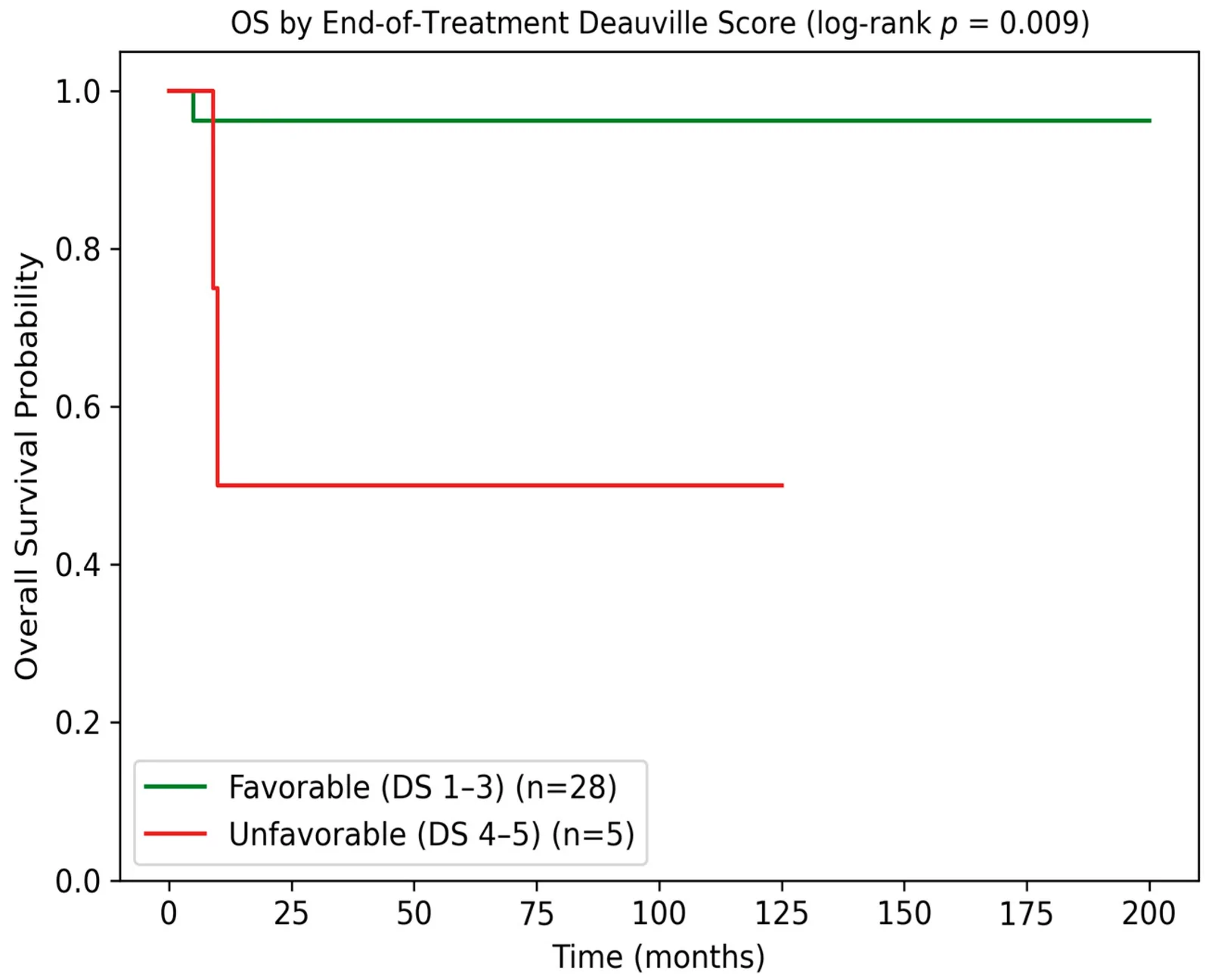
Overall survival by end-of-treatment Deauville score group (favorable, DS 1–3, vs. unfavorable, DS 4–5; log-rank *p* = 0.009).

Three-year and 5-year OS were both 89.6%, and 3-year and 5-year PFS were both 90.1% (median OS/PFS not reached; three events for each endpoint) (Figure 2). Patients with a favorable EOT DS (1–3) had significantly higher OS than those with an unfavorable DS (4–5) (96.4% vs. 60.0%; log-rank *p* = 0.009) (Figure 3). On univariate Cox regression, an unfavorable EOT DS was associated with markedly increased hazard for a PFS event (HR 13.10, 95% CI 1.18–145.1; *p* = 0.036), although the wide confidence interval reflects the small number of events. OS did not differ significantly between early- and advanced-stage disease (100% vs. 90.0%; log-rank *p* = 0.630).

### 3.6. Predictive Value of PET/CT Parameters

**Figure 4:**
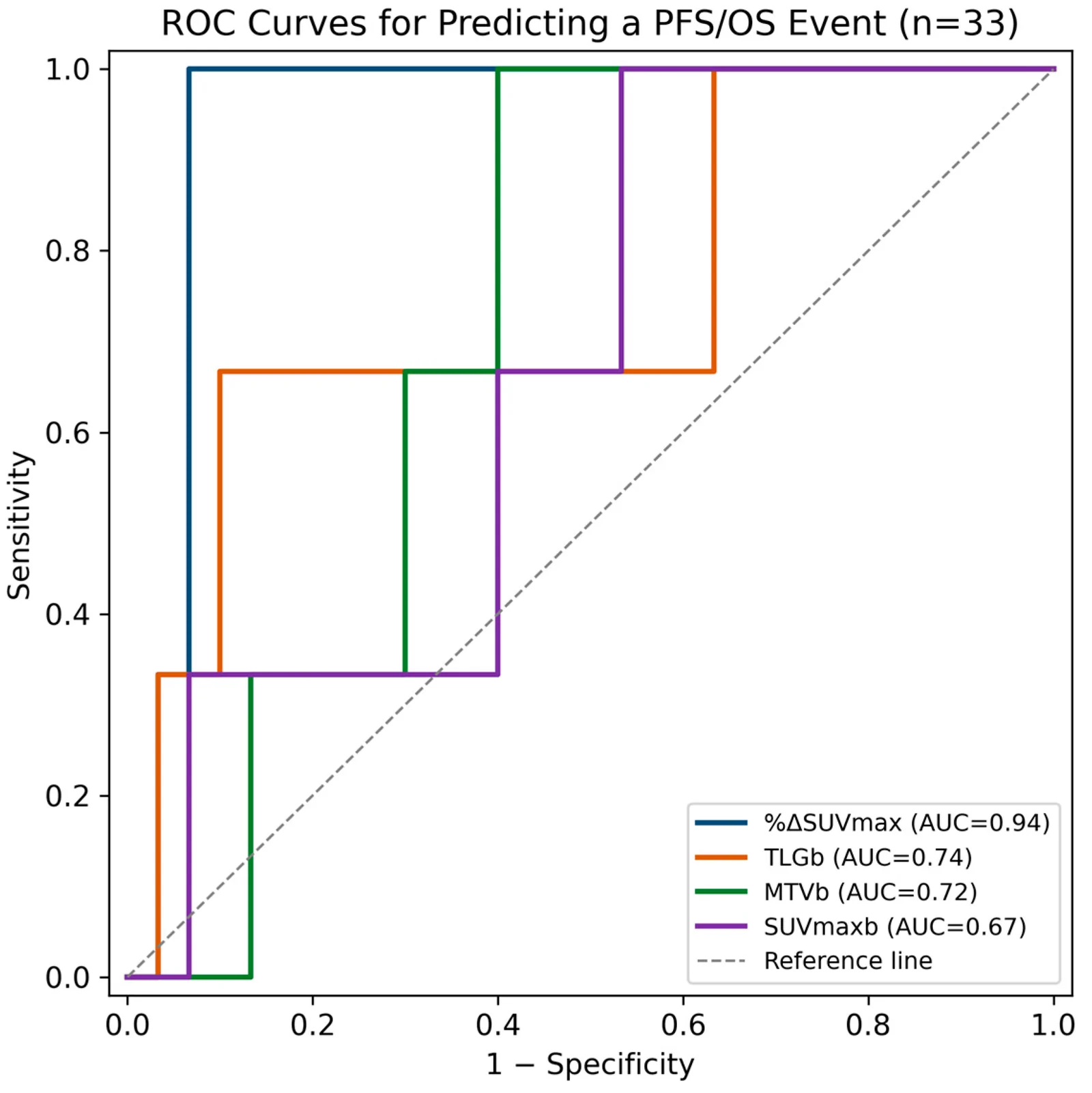
ROC curves for baseline SUVmax, MTV, TLG, and %ΔSUVmax (baseline to EOT) in predicting a PFS/OS event.

On ROC analysis, %ΔSUVmax from baseline to EOT was the strongest and most precisely estimated predictor of a PFS/OS event (AUC = 0.94, bootstrap 95% CI 0.84–1.00), with an optimal cutoff of ≥73.6% reduction (sensitivity 100%, specificity 93%). Baseline TLG (AUC = 0.74, 95% CI 0.52–1.00) and baseline MTV (AUC = 0.72, 95% CI 0.53–0.91) showed moderate point estimates but very wide confidence intervals; baseline SUVmax was weaker (AUC = 0.67, 95% CI 0.51–0.97), and %ΔTLG and %ΔMTV had limited predictive value (AUC = 0.58 and 0.54, respectively) (Table 4, Figure 4). On univariate Cox regression, %ΔSUVmax was a significant continuous predictor of PFS (HR 0.93 per 1-percentage-point increase in reduction, 95% CI 0.86–0.99; *p* = 0.033). Given that only three events were observed, a multivariate model combining these parameters could not be reliably estimated.

### 3.7. Interim PET/CT

**Figure 5:**
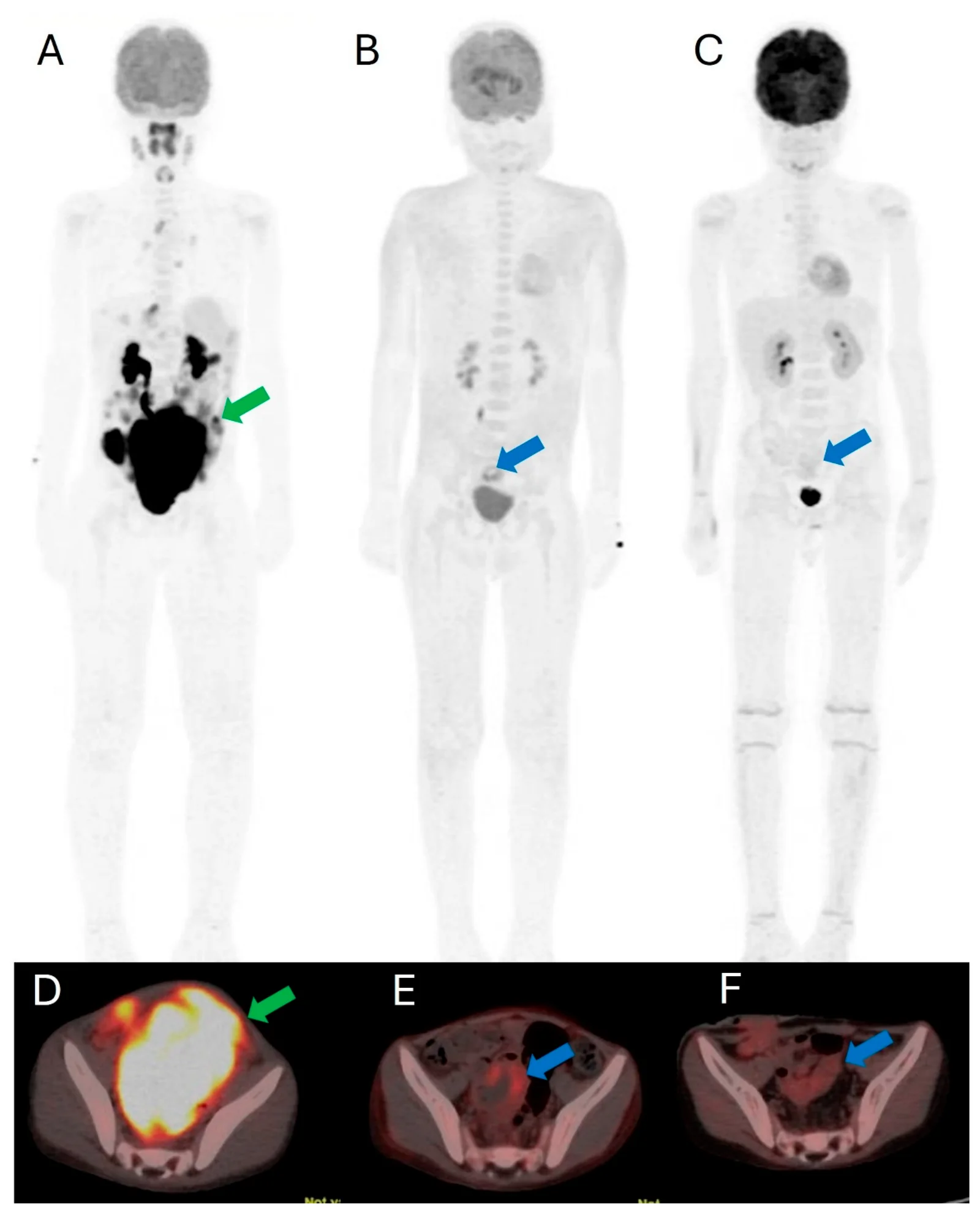
A 7-year-old boy with stage IV pediatric Burkitt lymphoma involving the abdomen and mediastinum, illustrating discordance between visual (Deauville) and quantitative (%ΔSUVmax) response assessment at the interim time point. (**A**) Baseline whole-body MIP showing an intensely hypermetabolic, bulky abdominal mass (green arrow; SUVmax 27.1, MTV 284.5 cm^3^, TLG 3474.6 g). (**B**) Interim whole-body MIP showing markedly decreased but still visually apparent residual uptake within the same region (blue arrow), corresponding to an interim Deauville score of 4 (unfavorable) despite an 81.9% reduction in SUVmax from baseline. (**C**) End-of-treatment whole-body MIP showing further reduction in residual metabolic activity (blue arrow), corresponding to a favorable end-of-treatment Deauville score of 3 and a 90.8% reduction in SUVmax from baseline. (**D**–**F**) Corresponding baseline, interim, and end-of-treatment axial fused PET/CT images. This case illustrates how a persistently positive visual Deauville score at the interim time point can co-occur with a quantitatively favorable metabolic response, supporting the potential complementary role of %ΔSUVmax alongside visual response criteria.

Interim PET/CT was available in 19 of 33 patients (57.6%). Interim DS was 2 in seven, 3 in five, and 4 in seven patients; no patient had an interim DS of 1 or 5. Twelve patients (63.2%) had a favorable interim DS (1–3) and seven (36.8%) an unfavorable interim DS (4–5). Within this subset, only two PFS/OS events occurred, both in the unfavorable interim DS group; 3-year OS and PFS were 100% in patients with a favorable interim scan versus 71.4% in those with an unfavorable scan (log-rank *p* = 0.045 for OS, *p* = 0.049 for PFS). On ROC analysis restricted to this subset, interim TLG showed the strongest discrimination for a PFS/OS event (AUC = 0.88), followed by the percentage reduction in SUVmax from baseline to interim (AUC = 0.79), interim SUVmax (AUC = 0.76), interim MTV (AUC = 0.74), and the percentage reduction in TLG from baseline to interim (AUC = 0.82); the percentage reduction in MTV from baseline to interim showed weak discrimination (AUC = 0.59). Given that only two events occurred within this 19-patient subset, these estimates are based on a very small number of outcomes and should be regarded as exploratory rather than confirmatory (Figure 5).

### 3.8. Sensitivity Analysis

Two patients (long-term survivors with unexpectedly high EOT MTV/TLG relative to the rest of the cohort) were excluded in a sensitivity analysis. Findings were materially unchanged: 3-year/5-year OS was 88.8% and PFS was 89.4% in the remaining 31 patients, and %ΔSUVmax remained the strongest predictor (AUC = 0.96). The log-rank comparison by EOT DS remained significant (*p* = 0.013).

## 4. Discussion

Rapid cell turnover and the resulting high FDG avidity make 18F-FDG PET/CT a natural fit for staging and response assessment in pediatric BL, yet quantitative prognostic data specific to children have been limited. In this retrospective cohort of 33 children, we found that the percentage reduction in SUVmax from baseline to EOT (%ΔSUVmax) was the strongest predictor of survival, and that an unfavorable EOT Deauville score was associated with significantly worse OS.

Our findings build on a small existing body of pediatric-specific work. Bailly et al. reported that a negative EOT DS carried a 93% negative predictive value and was associated with significantly higher 5-year PFS in 19 children with BL [14], and Xiao et al., [15] in a larger cohort of 68 pediatric patients, found that baseline tMTV and TLG, but not SUVmax, were independent predictors of PFS and OS on multivariate Cox regression. Parallel work in adult BL by Albano and colleagues demonstrated the prognostic value of baseline and dynamic 18F-FDG PET/CT parameters [12], including ΔMTV and ΔTLG [11], and showed that the EOT Deauville score and IHP criteria [13] were prognostically informative.

In our pediatric cohort, baseline SUVmax, MTV, and TLG showed only modest discriminative ability (AUC 0.67–0.74) and did not differ significantly by stage or splenic involvement, suggesting that static baseline measurements capture tumor burden but not treatment sensitivity. This contrasts with Xiao et al., where baseline tMTV and TLG were independent predictors with considerably larger effect sizes, and with Chen et al., who found baseline tMTV and TLG to be strong independent predictors of PFS and OS (AUC 0.82 for both) in a broader cohort of 46 pediatric patients with mature B-cell non-Hodgkin lymphoma, of which BL was a major subtype [24]. However, bootstrap 95% confidence intervals around our own baseline AUC estimates were wide (MTVb 0.53–0.91; TLGb 0.52–1.00), reflecting the small number of events (*n* = 3) rather than a precisely estimated null effect; these intervals comfortably include the point estimates reported by Xiao et al. and Chen et al. [15,24]. Our findings should therefore be read as inconclusive rather than contradictory regarding the prognostic value of baseline MTV and TLG in pediatric BL: we lack the statistical power to confirm or refute the associations reported in these larger cohorts, and the apparent discrepancy in point estimates may simply reflect sampling variability rather than a true difference in the underlying association. In contrast, our %ΔSUVmax estimate was both large and comparatively precise (AUC = 0.94, 95% CI 0.84–1.00), consistent with reports in other aggressive lymphomas, including DLBCL, where interval change in FDG uptake has outperformed baseline values as a prognostic marker, suggesting that this dynamic parameter may be a more robust signal even in a small cohort.

A plausible biological explanation for this divergence relates to what each parameter measures. Baseline MTV and TLG quantify the extent and intensity of disease at diagnosis, which in BL is characteristically high across nearly all patients given its near-uniform, extremely high proliferative index (Ki-67 approaching 100%); this ceiling effect may compress the dynamic range of baseline measurements and limit their ability to discriminate patients by underlying chemosensitivity before any treatment has been delivered. The %ΔSUVmax, by contrast, captures the tumor’s functional response to induction chemotherapy, a parameter more directly linked to the biological processes, such as apoptosis and cell kill, that ultimately determine outcome, and one that is less dependent on pretreatment tumor bulk. This distinction parallels observations in other FDG-avid, rapidly proliferating lymphomas, including DLBCL, where early metabolic response has repeatedly outperformed baseline metabolic burden as a prognostic marker. We propose this framework as a plausible explanation rather than an established mechanism, given the modest size of our cohort.

Interim PET/CT has shown limited and inconsistent prognostic value in prior BL series. In adult BL, Albano et al. found that interim DS did not predict PFS or OS, whereas the EOT scan was strongly predictive using the same criteria [14]. Consistent with this pattern, most published pediatric BL series, including those of Bailly et al. and Xiao et al., have centered their prognostic analyses on baseline or EOT PET/CT rather than interim imaging [15,16]. In our cohort, interim PET/CT was available in only 19 of 33 patients (57.6%), and within this subset only two PFS/OS events occurred. Although the interim DS grouping and interim metabolic parameters showed nominally significant or numerically strong associations with outcome (e.g., interim TLG AUC = 0.88), these estimates rest on an extremely small number of events and are likely to be highly unstable; we lacked sufficient statistical power in this subset to draw firm conclusions about the added value of interim imaging, and these findings should be considered exploratory. Larger studies with more complete interim PET/CT data collection are needed to determine whether interim response assessment adds meaningful prognostic information beyond the EOT scan in pediatric BL.

The EOT Deauville score remains a practical, widely available visual tool for response assessment. In our cohort, patients with a favorable score (DS1–3) had markedly better OS than those with an unfavorable score (DS4–5) (96.4% vs. 60.0%; log-rank *p* = 0.009) (Figure 3 and Figure 5). This finding should be interpreted with caution: only three deaths occurred during follow-up, and the corresponding Cox regression hazard ratio had a wide confidence interval (95% CI 1.18–145.1). While the direction and statistical significance of this association were consistent across two complementary methods (log-rank test and univariate Cox regression), and were unchanged as the cohort was expanded, the small number of events limits the precision of the estimate, and this result should be regarded as hypothesis-generating rather than confirmatory. Because of this limited number of events, we were also unable to fit a multivariate model combining Deauville score, %ΔSUVmax, and baseline parameters; larger, ideally multicenter or prospective, pediatric BL cohorts are needed to determine whether these visual and quantitative response criteria provide complementary or redundant prognostic information.

We did not identify a significant association between baseline MTV or TLG and stage or splenic involvement, in contrast to some studies in adult BL that have reported an association between higher baseline volumetric burden and advanced stage, and to broader pediatric lymphoma data in which metabolic tumor burden has tracked with disease extent [25]. This discrepancy may reflect the smaller sample size and the wide range of baseline tumor volumes observed in our pediatric cohort, in which extremely bulky disease was not confined to advanced-stage patients.

Although baseline MTV and TLG were not significantly associated with the St. Jude stage in our cohort, both were significantly higher in the ten patients with documented bone marrow involvement, consistent with bone marrow disease reflecting a particularly high systemic tumor burden. Bone marrow involvement was also associated with numerically lower 3-year OS and PFS and a Cox hazard ratio directionally consistent with worse outcome, although neither reached statistical significance given the small number of events. This pattern aligns with previous pediatric BL and mature B-cell NHL studies identifying bone marrow or CNS involvement as adverse prognostic factors [15,24]. Because bone marrow involvement is incorporated into stage IV disease, we could not fully disentangle its effect from that of advanced stage in this small cohort. In addition, serum LDH levels and CNS status, both established clinical indicators of disease burden and adverse prognosis in pediatric BL, were unavailable in our retrospective dataset. Higher LDH levels and CNS involvement may coexist with more extensive disease and, consequently, higher baseline MTV and TLG. Therefore, the observed relationship between metabolic tumor burden and outcome may partly overlap with these conventional clinical risk factors, and the independent prognostic contribution of baseline PET-derived volumetric parameters could not be determined in the present cohort.

This study has several limitations. First, its retrospective, single-center design and small number of survival events precluded multivariate survival modeling. Second, PET/CT examinations were acquired on two different scanner systems over the 16-year study period (Siemens Biograph TruePoint HD LSO (Siemens Healthineers, Germany) before 2019 and GE Discovery IQ (GE Healthcare, Milwaukee, WI, USA) from 2018 onward), with different reconstruction algorithms applied, which may have introduced variability in lesion detectability and quantitative PET measurements, including SUVmax, MTV, and TLG. Third, interim PET/CT was available for only 19 of 33 patients (57.6%), limiting our ability to fully characterize the added value of interim response assessment. Fourth, two patients showed an atypical combination of high EOT metabolic burden and long-term event-free survival; while a sensitivity analysis excluding these patients did not materially change our conclusions, we cannot fully exclude measurement or data-entry variability in these cases. Finally, given the rarity of pediatric BL, validation in an independent, ideally multicenter, cohort is needed before these thresholds can be applied clinically.

## 5. Conclusions

In children with Burkitt lymphoma, the percentage reduction in SUVmax from baseline to end-of-treatment PET/CT and the end-of-treatment Deauville score appear to be associated with survival outcomes, while baseline SUVmax, MTV, and TLG showed only modest prognostic value in our cohort. Although these findings should be interpreted cautiously because of the small number of survival events, they support further investigation of dynamic PET response metrics as potential tools for risk stratification in larger prospective pediatric studies.

## Figures and Tables

**Table 1:** Patient characteristics.

Characteristic	n (%)/Mean ± SD (Range)
Sex	Male 24 (72.7%) Female 9 (27.3%)
Age at diagnosis (years)	10.4 ± 4.7 (3–18)
Follow-up (months min–max)	median 44.0 mean 61.9 ± 58.1 (4–200)
Stage	Stage II: 3 (9.1%) Stage III: 10 (30.3%) Stage IV: 20 (60.6%)
Disease distribution	mixed 21 (63.6%) nodal 9 (27.3%) extranodal 3 (9.1%)
Splenic involvement	3 (9.1%)
Interim PET/CT performed (n)	19 (57.6%)
Vital status	Alive 30 (90.9%) Exitus 3 (9.1%)

**Table 2:** Baseline 18F-FDG PET/CT parameters, overall and by stage/splenic involvement.

Parameter	Overall (*n* = 33)	Early vs. Advanced Stage, *p*	Splenic±, *p*
SUVmaxb	17.9 ± 7.5 (4.5–37.4)	0.977	0.745
MTVb (cm^3^)	691.0 ± 820.4 (17.0–3309.7)	0.382	0.491
TLGb (g)	4600.0 ± 4196.5 (40.5–17,370.0)	0.348	0.382

**Table 3:** Baseline and end-of-treatment 18F-FDG PET/CT parameters.

Parameter	Baseline	End-of-Treatment
SUVmax	17.9 ± 7.5 (4.5–37.4)	2.5 ± 0.9 (0.5–5.0)
MTV (cm^3^)	691.0 ± 820.4 (17.0–3309.7)	87.0 ± 163.7 (1.3–735.8)
TLG (g)	4600.0 ± 4196.5 (40.5–17,370.0)	161.8 ± 351.0 (1.9–1602.6)
% reduction, baseline–EOT	—	SUVmax 83.3 ± 10.7% MTV 82.9 ± 15.2% TLG 95.5 ± 9.4%

**Table 4:** ROC analysis of baseline and percentage-change PET/CT parameters for predicting a PFS/OS event. Optimal cutoffs, sensitivity, and specificity are not reported for %ΔTLG and %ΔMTV given their AUC values close to chance (≤0.6).

Parameter	AUC	Optimal Cutoff	Sens.	Spec.
%ΔSUVmax (b–EOT)	0.94	≥73.6% (reduction)	100%	93%
TLGb	0.74	≥8948 g	67%	90%
MTVb	0.72	≥404 cm^3^	100%	58%
SUVmaxb	0.67	≥19.1	100%	47%
%ΔTLG (b–EOT)	0.58	—	—	—
%ΔMTV (b–EOT)	0.54	—	—	—

## Data Availability

The data presented in this study are available on request from the corresponding authors, due to institutional and patient privacy restrictions.
